# Oxidative RNA Damage in the Pathogenesis and Treatment of Type 2 Diabetes

**DOI:** 10.3389/fphys.2022.725919

**Published:** 2022-03-28

**Authors:** Xiatian Chen, Hua Yu, Zhe Li, Wei Ye, Ziqian Liu, Jinning Gao, Yin Wang, Xin Li, Lei Zhang, Natalia Alenina, Michael Bader, Hongyan Ding, Peifeng Li, Lynn Htet Htet Aung

**Affiliations:** ^1^ Center for Molecular Genetics, Institute of Translational Medicine, The Affiliated Hospital of Qingdao University, College of Medicine, Qingdao University, Qingdao, China; ^2^ School of Basic Medicine, Qingdao University, Qingdao, China; ^3^ The Affiliated Cardiovascular Hospital of Qingdao University, Qingdao, China; ^4^ Jiangsu Provincial Engineering Research Center for Biomedical Materials and Advanced Medical Device, Huaiyin Institute of Technology, Huaian, China; ^5^ Max-Delbrück-Center for Molecular Medicine (MDC), Berlin, Germany; ^6^ School of Bioengineering, Suqian University, Suqian, China

**Keywords:** oxidative damage, RNA oxidation, 8-oxoGuo, type 2 diabetes, therapeutic strategy

## Abstract

Excessive production of free radicals can induce cellular damage, which is associated with many diseases. RNA is more susceptible to oxidative damage than DNA due to its single-stranded structure, and lack of protective proteins. Yet, oxidative damage to RNAs received little attention. Accumulating evidence reveals that oxidized RNAs may be dysfunctional and play fundamental role in the occurrence and development of type 2 diabetes (T2D) and its complications. Oxidized guanine nucleoside, 8-oxo-7, 8-dihydroguanine (8-oxoGuo) is a biomarker of RNA oxidation that could be associated with prognosis in patients with T2D. Nowadays, some clinical trials used antioxidants for the treatment of T2D, though the pharmacological effects remained unclear. In this review, we overview the cellular handling mechanisms and the consequences of the oxidative RNA damage for the better understanding of pathogenesis of T2D and may provide new insights to better therapeutic strategy.

## Introduction

Free radicals are the normal cellular products of energy metabolism (Kong and Lin, 2010). In living organisms, radicals are mostly derived from reactive oxygen species (ROS) and reactive nitrogen species (RNS). However, excessive production of free radicals may cause mitochondria dysfunction, which could lead to the changing of energy metabolism and affect the normal equilibrium of cells (Valko et al., 2007). Free radicals can damage the DNAs, RNAs and proteins as well as other cell components (Haigis and Yankner, 2010; Majzunova et al., 2013; McArdle and Jackson, 2019). Oxidative stress occurs when free radicals are over-produced or due to the decrease in the ability of the antioxidant defense system. Indeed, oxidative damage exists widely in the body, which is mainly manifested by the damage to the structure and function of biological macromolecules (such as DNA, proteins, lipids, etc.), which leads to gene mutation, cell carcinogenesis, and aging. The pathogenesis of some age-related diseases such as neurological disorders (i.e., Alzheimer’s disease, amyotrophic lateral sclerosis, Parkinson’s disease) and atherosclerosis have also been attributed to the oxidation of the chemicals induced by free radicals (Poulsen et al., 2012; Fimognari, 2015).

Nowadays, noncommunicable diseases (NCDs), including diabetes, cancer, and cardiovascular diseases, are the major cause of death globally. NCDs account for 63 percent of all deaths worldwide every year. Diabetes is one of the most essential NCDs that threatening human health in the world, the rapid growth of diabetes has brought an alarming global burden to the social and economic development (Jaacks et al., 2016). The international diabetes federation (IDF) reported that the number of diabetes patients could reach over six hundred million by 2040 (Ogurtsova et al., 2017; Luo et al., 2019). In diabetes, Type 2 diabetes (T2D) constitutes the main type (Ogurtsova et al., 2017). T2D could cause a series of concomitant diseases, such as coronary heart disease, lower limb arteriopathy, retinopathy, diabetic nephropathy (Sung et al., 2018).

And now, there are many strategies for the treatment of T2D, mainly includes surgery, drug therapy, exercise therapy, dietary nutrition, and multifactorial treatment (Durrer Schutz et al., 2019; Koch and Shope, 2021). However, due to the diversity of disease factors in T2D, these methods cannot effectively reduce the increase in morbidity and mortality. In addition, the level of venous blood glucose and biomarkers like glycated hemoglobin (HbA1c) also have limitations to be used as prognostic and predictive biomarkers for mortality, especially for elders or patients with hyperlipidemia (Durrer Schutz et al., 2019). Therefore, much work remains to be done to understand the underlying mechanism involved in the regulation of glucose metabolism and insulin sensitivity, which will unveil a better understanding of blood glucose homeostasis and find more potential therapy targets.

In this review, we discuss the consequences and the mechanism of intracellular response to RNA oxidation. Furthermore, we review the relationship between the ROS and T2D which may provide new intervention strategy for T2D.

## The History of RNA Functions and Structure

Although F. Miescher discovered the nuclein in 1868, it did not attract much attention for more than half a century (Sankaran, 2016). In the 1920s and 1930s, it was confirmed that there were two kinds of nucleic acids DNA and RNA in nature, and the composition of nucleotides was clarified. In the decades that followed, life sciences focused on the application and analysis of the genomic DNA. In the 1980s, W. Gilbert et al. discovered that RNA had the activity of catalytic enzymes, which opened a window for the study of RNA (Sankaran, 2016). In addition to transferring information from DNA to protein, RNA molecules also play vital role in maintaining, regulating, and protecting the genomes of all organisms and have more structural and functional diversity than DNA (Sharp, 2009; Cech and Steitz, 2014).

Biomolecules have unique molecular structure, which is essential for their function. However, we know very little about the structure of RNA. The exploration of RNA structure started from the Click coined the hypothesis that RNA played an intermediary role in the transmission of genetic information to proteins in 1953. And in 1965, Holly and his colleagues used nuclease to sequence yeast tRNA (Holley et al., 1965). Noller and Chaires found that kethoxal modifies affected the function of rRNA in 1972 (Noller and Chaires, 1972). Subsequently, the studies focus on the secondary and tertiary structures of RNA (Peattie and Gilbert, 1980). High-throughput sequencing and new computational methods to interrogate RNA structure are beginning to provide new insights into RNA structures (Kertesz et al., 2010; Underwood et al., 2010; Lucks et al., 2011). Driven by technology and cognition, more and more structural changes, modifications, and types of RNA have been gradually discovered. At the same time, the remarkable diversity of RNA structure and function revealed an enormous potential of RNA in future clinical applications is gradually emerging.

## The Generation and Damage of ROS in T2D

The theory of ROS in organisms was put forward in the 1950s (Commoner et al., 1954; Newsholme et al., 2016). ROS can be produced by exogenous and endogenous sources (Kohen and Gati, 2000). Exogenous sources may include ultraviolet radiation (Liu et al., 2020), ultrasound, drugs, radiation and exposure to pollutants and toxic chemicals. Endogenous sources may include neutrophils, cytokines (Obata et al., 2001), enzymes that directly produce ROS. These enzymes mainly come from mitochondria, such as dihydroorotate dehydrogenase, succinate dehydrogenase and NADPH oxidases (NOX) (Le Bras et al., 2005; Ahmad et al., 2017). Among those enzymes, NOX is an enzyme that localizes on cell membrane and catalyzes the reduction of oxygen molecules to superoxide anion (Dubois-Deruy et al., 2020; Masselli et al., 2020). It is the main source of ROS generation. As a coenzyme, NOX participates in cellular electron transfer and servers as a second messenger in many signaling pathways (Bae et al., 2011).

**FIGURE 1:**
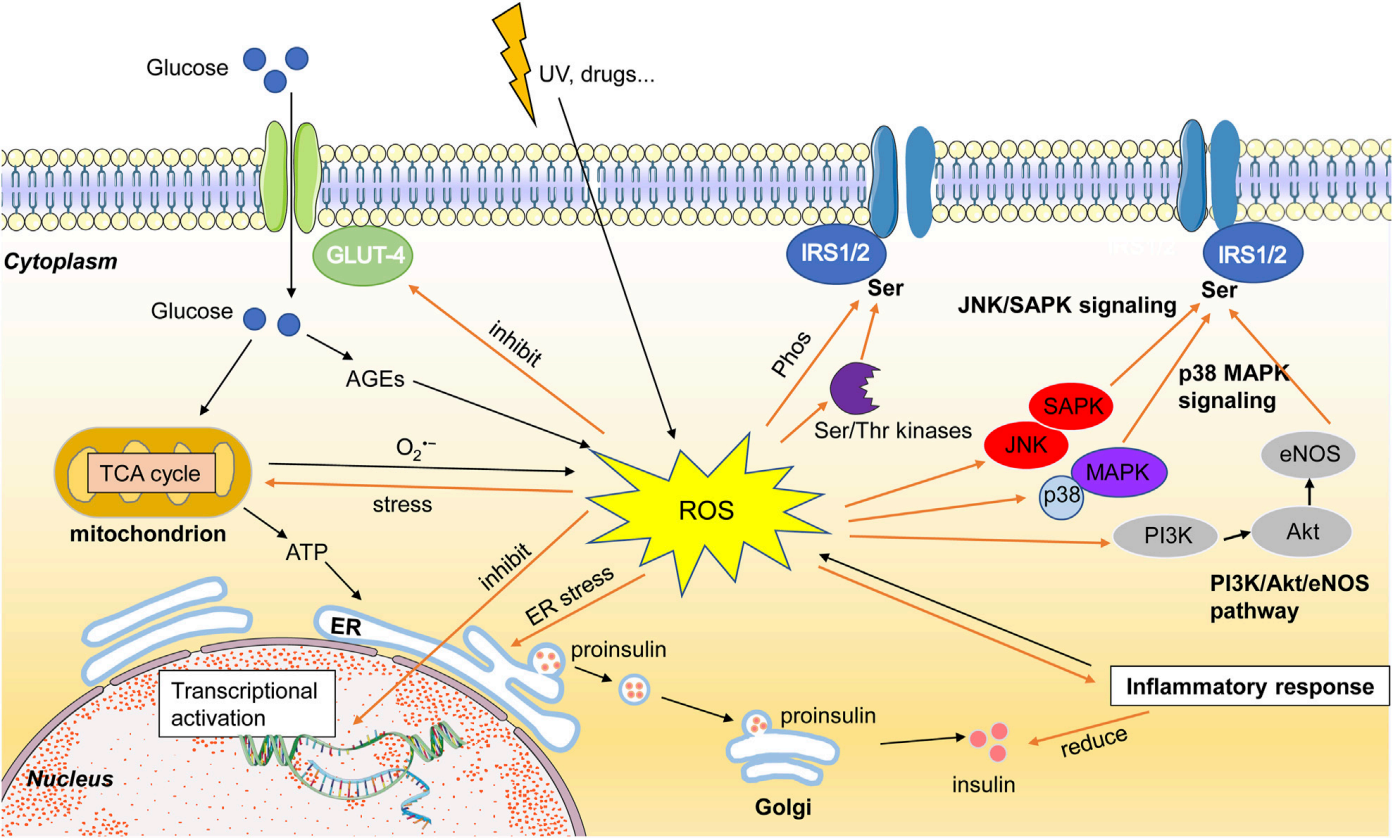
Schematic diagram of the effect of ROS toxicity on insulin secretion and insulin resistance signaling pathways. High level of ROS can modulate insulin resistance by altering the insulin-signaling ways, such as Akt, P13K, and JNK. ROS also can inhibit the function of cell organelles, and then reduce the energy supply, induce the apoptosis.

ROS have been reported to contributed the pathology of T2D in many ways (Figure 1). High glucose intake in the body increases mitochondrial production of ATP, which produces large amounts of free radicals and increases ROS levels. And excess sugar can bind to proteins to form advanced glycation products (AGEs).

Accumulating studies demonstrated that ROS plays a vital role in the occurrence and development of T2D by inducing insulin resistance and beta cell dysfunction (Rochette et al., 2014; Liu et al., 2016). A classical mechanism of insulin abnormality is dysregulation of the insulin-regulated pathway, such as inappropriate phosphorylation or inhibition of the insulin receptor-associated proteins. The insulin binds to insulin receptors will lead to phosphorylation of residues, resulting in changes in receptor structure and triggering downstream signal transduction. Excess free radicals can disturb beta cell function, inhibiting proliferation and regeneration (Porte and Kahn, 2001; Liang et al., 2016; White et al., 2016; Wang and Wang, 2017).

The insulin receptor substrate (IRS) proteins are the cytoplasmic proteins that regulate growth and metabolism, which response to some stimuli including hormones and cytokines (Lee and White, 2004). The family of IRS was compound of six type named IRS-1, IRS-2, IRS-3, IRS-4, IRS-5, and IRS-6. Among them, IRS-1 and IRS-2 are the key control of IR (Copps and White, 2012). ROS could induce IR by leading the phosphorylation of the serine at serin 307 of IRS-1. Excess free radicals could also reduce the migration and relocation of insulin-dependent IRS (Yaribeygi et al., 2019). The high level of ROS can also impair the insulin sensitivity by activating the IKKβ/NF-κB and JNK pathway, resulting the degradation of IRS-1/2 (Nishikawa et al., 2000; Evans et al., 2005). ROS-mediated activation of JNK signaling leads to decreased insulin secretion *via* nucleocytoplasmic translocation of pancreatic and duodenal homeobox-1 (PDX-1), a key transcription factor that binds to the insulin promoter and induces insulin expression (Kajimoto and Kaneto, 2004). High levels of intracellular oxidation can also inhibit p38 MAPK pathway, PI3K/Akt/eNOS pathway, PI3K/JUK pathway, JNK/SAPK pathway, modulate serine/threonine kinases such as Akt (or PKB), GSK-3, AMPK, and mTOR, which cause IR (Bloch-Damti and Bashan, 2005; Rains and Jain, 2011; Yaribeygi et al., 2020).

Glucose transporter-4 (GLUT-4) have the function of transporting the glucose into cells, any factor that reduces the expression of GLUT-4 have a significant effect on the insulin signal (Klip et al., 2019). Clinal researches showed the expression of GLUT-4 was lower than the control in the patients with insulin resistance (Hussey et al., 2012; Reno et al., 2017). ROS could suppress the expression and relocation of GLUT-4 by inducing the point mutation (Hurrle and Hsu, 2017). And some studies reported that ROS also could suppress the GLUT-4 expression by effecting the expression of its transcriptional factors such as PPAR-γ, CEB/Ps, nuclear factor-1, p85, HIF-1α, MEF2, and NF-κB (Cooke and Lane, 1999; Pessler et al., 2001; She and Mao, 2011).

ROS is mainly derived from mitochondria, but can also in turn lead to mitochondrial stress, mitochondrial dysfunction, mitochondrial division, and cell apoptosis (Medda et al., 2021). As noted above that the mitochondrial dysfunction can affect the energy supply and indirectly affect insulin secretion.

Endoplasmic reticulum (ER) is the main organelle for intracellular modification and synthesis. The stimuli of oxidative stress could disturb the function of ER and induce the ER stress (Lemmer et al., 2021). ER stress could disrupt proper protein folding leading to accumulation of misfolded proteins, which can lead to reduced insulin synthesis and decreased insulin stability in beta cells (Han et al., 2013). ER stress can suppress the expression of GLUT-4 and relocation on the cell membrane (Yang et al., 2010). Ozcan et al. (2004) reported the ER stress could induce IR by directly suppressing the insulin pathway through hyperactivation of c-Jun N-terminal kinase (JNK) and subsequent serine phosphorylation of IRS-1 (Nakatani et al., 2005). The accumulation of misfolded proteins in ER stress may cause the unfolded protein response (UPR), which will promote the cell death during the prolong stress (Fernández et al., 2015).

Oxidative stress stimulates the inflammatory response, which in turn worsens oxidative levels, and numerous studies have shown that oxidative stress and inflammation are key factors in the development of insulin resistance and the establishment of T2D (Keane et al., 2015). ROS could increase inflammatory responses which in turn induces insulin resistance in several pathways, such as IKK, JNK and NF-κB signaling pathway (Cai et al., 2005), IL-6, TNF-a/JNK/IRS-1 pathway (Akash et al., 2018).

## The Indicator of RNA Oxidation: 8-oxoGuo

Oxidation of RNA could cause strand breaks, lose the expression of RNA, and then induce modified nucleobases, lipid, and sugar (Li et al., 2006; Jacobs et al., 2010; Resendiz et al., 2012). The oxidation products of RNA mainly include 5-hydroxycytidine, 5-hydroxyuridine, 8-hydroxyadenosine and 8-oxoGuo. Among various oxidation formations, the oxidized nucleobase 8-oxoGuo in RNA is currently used as an essential indicator of RNA oxidation because it appears to be abundant and mutagenic (Li et al., 2006).

**FIGURE 2:**
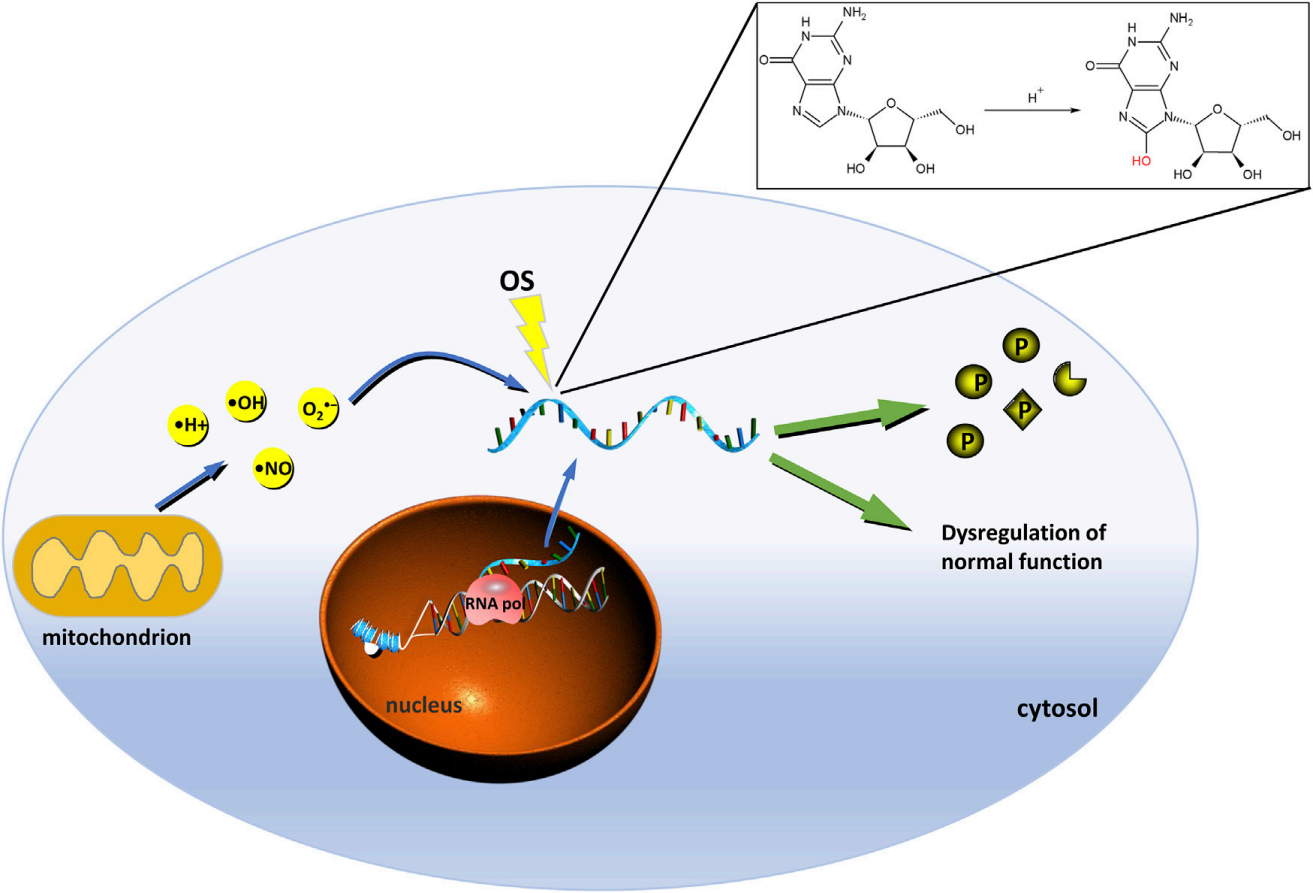
Mode of the occurrence and consequence of RNA oxidation. Free radicals attacked the eighth carbon atom of RNA to turn G into 8-oxoGuo. Once RNA is oxidized, the original function would become abnormal. Oxidized coding RNAs lead to decrease in the level and activity of protein synthesis and increase the non-functional, incomplete protein, and mutated proteins. Oxidation of non-coding RNA could affect their regulatory functions. The green marked P ball represents a normal protein, the notched sphere indicates non-functional or truncated protein. Rhombohedral sphere indicates mutated protein. RNA pol, RNA polymerase; P, protein; OS, oxidative stress.

8-oxoGuo is an oxidative adduct produced by reactive oxygen radicals such as hydroxyl radicals and singlet oxygen that attack the carbon 8 of the guanine base in RNA molecules (Figure 2). The degree of oxidative damage and repair *in vivo* can be evaluated by the detection of 8-oxoGuo, and the relationship between oxidative stress and RNA damage (Schöttker et al., 2020). It is of great significance for the study of degenerative diseases, aging mechanism, carcinogenesis mechanism, the relationship between environmental toxins and oxidative stress. It can also be used to evaluate the efficacy of antioxidants in the treatment of RNA oxidative damage.

As mentioned above, DNA was discovered and studied first, and most previous studies were focus on the DNA oxidation. The first report to assessed the level of 8-hydroxydeoxyguanosine in urine was in 1962 (Loft et al., 1992). Franzke et al. (2018) found that exercise, nutrition and cognitive training increased antioxidant activity by measuring oxidative markers in blood plasma. Larsen et al. (2019) pointed that clinical study must be considered the study population and size, as many factors such as environment can contribute to differences.

Several methods have been reported to detect RNA oxidation levels. However, due to the lack in the RNA oxidation studies, the methods need to be developed and updated. Nowadays, the measure of oxidized RNA products is based on the chemical structure or immune properties of oxidized nucleotides. Nucleosides and bases could be detected by high-performance liquid phase chromatography coupled with tandem mass spectrometry (HPLC–MS/MS) or ultraperformance liquid chromatography-tandem mass spectrometry (UPLC-MS) (Fiala et al., 1989; Shen et al., 2000; Hofer et al., 2006). Another detection method relies on the interaction of antigens and antibodies, including dot blot, ELISA, Northwestern blot, immunoprecipitation, and immunohistochemistry (Nunomura et al., 1999; Honda et al., 2005; Shan and Lin, 2006; Looi et al., 2008; McKinlay et al., 2012). Elisa methodology is one of the earliest, most widely used and relatively mature detection methods, which benefit from the reaction between antibodies (Cooke et al., 2001; Chiou et al., 2003). At present, there are a lot of kits on the market to detect oxidized nucleic acid are based on this principle (Chao et al., 2021). HPLC/UPLC-MS are relatively mature methods for the detection of RNA oxidation products (Wang et al., 2017; Jorgensen et al., 2018; Jørs et al., 2020). These methods have high accuracy while requiring many samples. ELISA and other biochemical reaction methods can detect components in body fluids, but the operation is complex and time-consuming. Since there is only one oxygen atom difference between 8-oxoGuo and G in structure, it is a challenge for the specificity of antibodies (Barregard et al., 2013). It is impossible for DNA or RNA specific recognition antibodies to reach all oxidative damage sites in nucleic acids, which could affect the quality of experimental data.

Even though there are many methods to detect the content of 8-oxoGuo in different samples, it is still quite challenging to measure it accurately. Because it will generate the uncontrollable new oxidation reactions during nucleic acid extraction and subsequent operations (Barregard et al., 2013). In order to increase the accuracy of the results as much as possible, metal chelation or antioxidants were added during the experiment to decrease the false new RNA oxidative damage during the experiment (Hofer et al., 2005). In addition, because the RNA oxidation could impede reverse transcription, the rate of reverse transcription is also used to assess the level of RNA oxidation (Gong et al., 2006).

Further studies reported that oxidation did not affect the association of RNA and polysome in the process of transcription, but affect the properties of the product (Shan et al., 2003; Shan et al., 2007). Oxidized coding RNAs lead to a decrease in the level and activity of protein synthesis and increase the non-functional, incomplete protein, and mutated proteins (Figure 2). Oxidized non-coding RNAs can inhibit their regulatory functions in the process of protein synthesis, leading to cell apoptosis and cell death. For instance, the microRNAs (miRNAs) are crucial member of non-coding RNAs and have been recognized as the vital regulator in the gene expression (Saliminejad et al., 2019; Kozak et al., 2020). MiR-184 upon oxidative modification could mismatch the 3′UTR of Bcl-xL and Bcl-w resulting in their reduction and this mismatch is involved in the initiation of apoptosis *in vitro* and vivo (Wang et al., 2015). Oxidized miR-1 could redirect the new targets in heart hypertrophy pathways that influence the redox mediated gene expression (Seok et al., 2020).

## Coping With the RNA Oxidation

**FIGURE 3:**
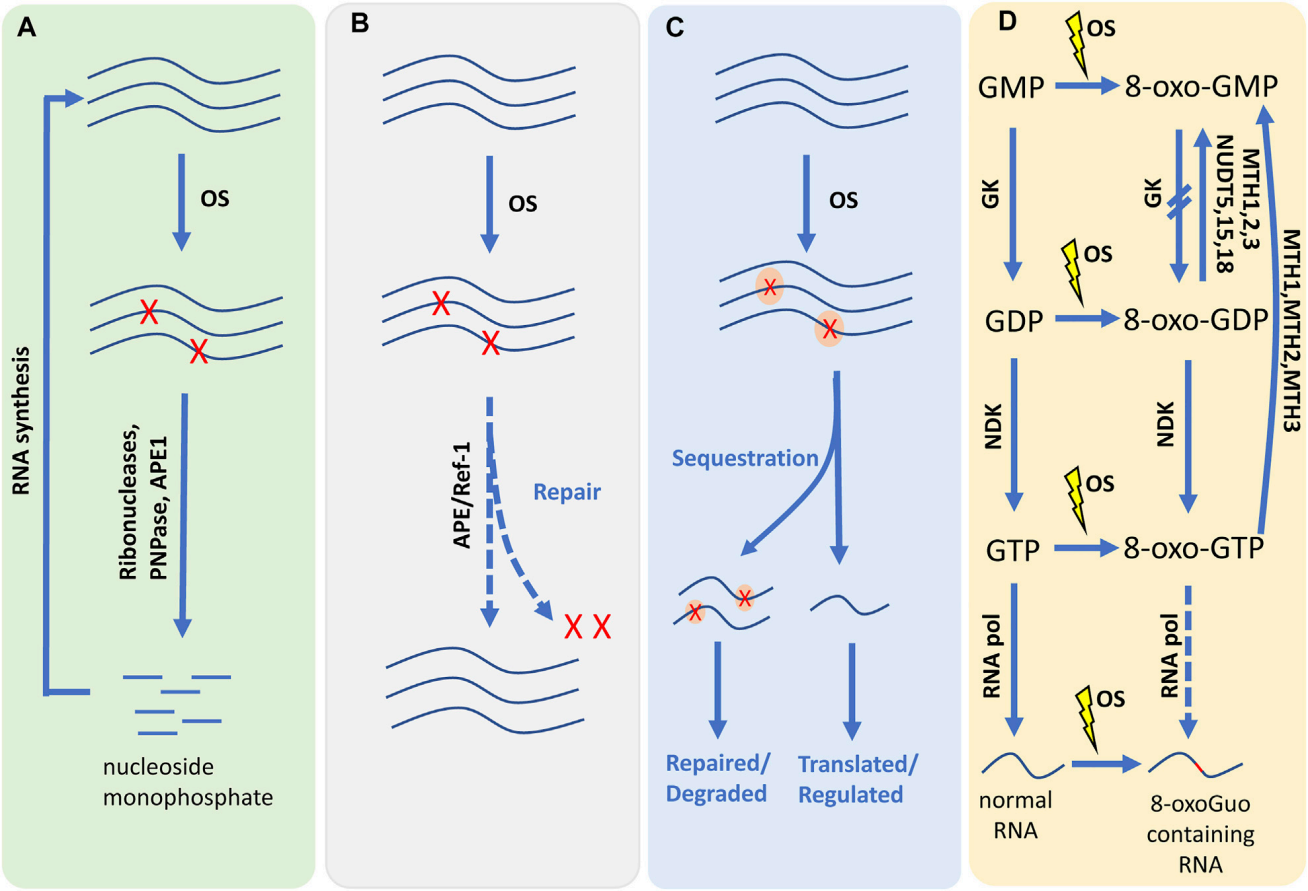
The schematic diagram of coping with RNA oxidation. **(A)** The oxidized RNA may lose its normal function, it would be degraded into nucleoside monophosphate by ribonucleases, PNPase or APE1. The nucleoside monophosphate can be reused in RNA synthesis. PNPase: Polynucleotide phosphorylase; APE1: Apurinic/apyrimidinic endonuclease 1. **(B)** The pathway of RNA repair has not been reported yet, but there are many studies speculating that it exists. Under the action of some substances, the oxidative damage of RNA will be repaired to perform its normal function. **(C)** Oxidized RNA molecules can be labeled with specific binding proteins. Once identified, oxidized RNA can be separated from normal RNA. Sequestration may help recruit repair/degradation activities that will eventually eliminate oxidized RNA. The orange ovals indicate binding proteins. **(D)** 8-oxoGuo containing RNA can be generated by oxidizing RNA and a few can be synthesis by 8-oxo-GTP. 8-oxo-GTP can be generated by oxidation of GTP as well as by phosphorylation of 8-oxo-GDP by NDK. 8-oxo-GDP can be generated by oxidation of GDP as well as by phosphorylation of 8-oxo-GMP by GK. MTH1, MTH2, MTH3 could hydrolyze 8-oxo-GDP and 8-oxo-GTP to 8-oxo-GMP. NUDT5, NUDT15, NUDT18 could hydrolyze 8-oxo-GDP to 8-oxo-GMP. GK: Guanylate kinase; NDK, nucleotide diphosphate kinase; MTH1, MutT homologue 1; MTH2, MutT homologue 2; MTH3, MutT homologue 3; NUDT5, Nudix type 5; NUDT15, Nudix type 15; NUDT18, Nudix type 18. OS, oxidized stress; The red blot in the 8-oxoG containing RNA indicates the oxidized nucleotide. Blue lines represent RNA molecules. Red “X” represents oxidized residues in RNA.

Living organisms need to reduce the risk of damage mediated by RNA oxidation. The cells must have some protective mechanisms to maintain the normal function and to survive under stress conditions, such as monitoring and inhibiting the process of RNA oxidation or keeping and reducing the excess oxidation molecules. Unfortunately, little is known about any of the mechanisms. Therefore, we discuss the progress and hypothesize potential ways in which oxidized RNAs may be involved in regulation (Figure 3).

### Degradation

Degradation can play an important role in RNA metabolism and eliminating oxidized RNA. This irreversibly eliminating pathway is probably the major system dealing with oxidized RNA. Ribonucleases have the degradation activity for oxidized RNA, and it can eradicate aberrant RNAs. However, further studies show that RNase is usually assisted by polyadenylation and RNA helicase activity (Deutscher, 2006). Polynucleotide phosphorylase (PNPase) is widely distributed across organisms of all kingdoms (Das et al., 2011). In *E.coli*, PNPase could regulate many aspects of RNA metabolism, including degradation of defective rRNA and tRNA, etc. (Kushner, 2002; Basturea et al., 2011). Human PNPase (hPNPase) is mainly localized in mitochondrial intermembrane space (Piwowarski et al., 2003; Chen et al., 2006). In human mitochondria, hPNPase and RNA helicase Hsuv3 form a complex, which maintains the homeostasis of some mt-mRNAs. And in cell culture, the hPNPase plays a vital role in the degradation of mitochondrial mRNA, c-myc RNA, and miRNAs. Several studies reported Apurinic/apyrimidinic endonuclease 1 (APE1) could control the RNA quality and degrade the oxidized RNA (Li et al., 2014).

### Repair

At present, very little is known about the repair mechanisms of oxidative RNA damage. It is very costly for cells to synthesize RNAs because those pathways require energy. If the damage to RNA is sublethal and can be repaired and reused, it could save cells energy and be evolutionarily beneficial. Although RNA is like DNA, RNA lacks a complementary strand; RNA-repair pathways may be different from the DNA repair mechanisms. Indeed, some studies have confirmed that RNA damage can be repaired. For instance, the methyl-guanine-methyl transferases (MGMT), which repair O^6^-mG (Kaina et al., 2007), and the oxidative demethylases, such as A1KB, repair m^1^A and m^3^C (Dinglay et al., 1998; Aas et al., 2003; Fu et al., 2010). The DNA-repair enzyme APE/Ref-1 has rRNA quality control (Fu et al., 2010).

### Sequestration

The oxidized RNA may lose its original biological function, some specific factors could discriminate and mark oxidized nucleotides during RNA synthesis or translation (such as RNA polymerase, etc.). And then, it will activate the recruitment of repair enzymes or degradation reactions (Deutscher, 2006). Several proteins have been demonstrated to recognize and bind oxidized RNAs. For instance, PNPase protein or hPNPase, which has a higher binding affinity to 8-oxoGuo than other normal oligonucleotides (Hayakawa et al., 2001; Hayakawa and Sekiguchi, 2006; Wu and Li, 2008). Mammalian Y box-binding protein 1 (YB-1), heterogeneous nuclear ribonucleoprotein D0 (HNRNPD), splicing factor 3B subunit 4 (SF3B4), heterogeneous nuclear ribonucleoprotein C1/C2 (HNRNPC) and splicing isoform 1 of DAZ-associated protein 1 (DAZAP1) also show affinity comparable to hPNPase protein (Hayakawa et al., 2002; Hayakawa et al., 2010).

### Interdicting Incorporation of Oxidized Nucleotides Into RNA

Oxidized nucleotides triphosphates from the nucleotide pools can be metabolically incorporated into RNA through the synthesis pathway, and oxidized nucleotides can be produced by degrading oxidized RNAs (Li et al., 2006). Under oxidative stress, nucleosides and nucleotides may be oxidized. RNA polymerase could discriminate guanosine-5′-triphosphate (GTP) and 8-oxo-7,8-dihydroguanosine-5′-triphosphate (8-oxo-GTP) during the process of RNA synthesis. The rate of misincorporation of 8-oxo-GTP into RNA was only 2% that of guanine (Hayakawa et al., 1999). 8-oxo-7,8-dihydroguanosine 5′-monophosphate (8-oxo-GMP) can be transformed into 8-oxo-7,8-dihydro-guanosine 5′-diphosphate (8-oxo-GDP) under the catalysis of guanylate kinase (GK), similarly, 8-oxo-GDP can be phosphorylated to 8-oxo-GTP through the nucleotide diphosphate kinase (NDK) (Ishibashi et al., 2005; Sekiguchi et al., 2013). Several enzymes can dephosphorylate oxidized nucleoside triphosphates, thus preventing their incorporation into RNA, have been identified. MutT homologue 1 (MTH1), MTH2, and MTH3 can hydrolyze 8-oxo-GDP or 8-oxo-GTP to 8-oxo-GMP (Hayakawa et al., 1999; Ishibashi et al., 2005; Takagi et al., 2012). Nudix type 5 (NUDT5), NUDT15, NUDT18 can hydrolyze 8-oxo-GDP to 8-oxo-GMP (Ishibashi et al., 2005). And GK could also block the phosphorylation of 8-oxo-GMP to 8-oxo-GDP (Li et al., 2014).

## RNA Oxidation and T2D

Many studies reported that T2D is associated with 8-oxoGuo. The first research about the relationship of RNA oxidation marker 8-oxoGuo and T2D was reported in 2011. Broedbaek and his colleagues analyzed approximately fourteen hundred diagnosed T2D patients and found that the level of 8-oxoGuo was positively correlated with the mortality of patients (Broedbaek et al., 2011). Moreover, Broedbaek confirmed the association between 8-oxoGuo and the mortality of T2D in the same cohort (Broedbaek et al., 2013). Besides, the levels of 8-oxoGuo were independent of other risk factors. Those findings suggest that the 8-oxoGuo could serve as a new clinical biomarker in diabetes.

Later, some studies reported that high RNA oxidation is associated with the risk of death in patients with all-cause, cardiovascular and microalbuminuria in patients with T2D (Kjær et al., 2017; Kjaer et al., 2018), and the imbalanced redox system could be a molecular mechanism contributing to the progression of T2D (Poulsen et al., 2019; Schöttker et al., 2020). RNA oxidation occurs earlier than DNA oxidation and is more closely associated with diabetic nephropathy. 8-oxoGuo may represent a new and easily detectable biomarker for diabetic nephropathy (Poulsen et al., 2019; Schöttker et al., 2020). Interestingly, this association of 8-oxoGuo in T2D was weak in younger patients, likely due to the higher tolerance to oxidative stress in youth (Schöttker et al., 2020).

The etiology of T2D is complicated. Currently, the main drugs include glipizide, biguanide, thiazolidinedione, and other oral drugs, as well as insulin and insulin-like injection preparations, were reported to decrease the level of oxidation. Long-term clinical follow-up studies have found that taking anti-hypertensive drugs such as losartan, valsrtan, and olmesartan could reduce DNA oxidation markers compared with the placebo (Kuboki et al., 2007; Ogawa et al., 2009; Nakamura et al., 2013; Pan et al., 2015).

Interestingly, in the therapeutic aspect of RNA oxidation, Broedbaek and his colleagues found that the usage of lipid-lowering drugs was associated with lower RNA oxidation, which could be the potential therapeutic drugs (Broedbaek et al., 2011). Another study reported that in T2D patients, a decrease of 8-oxoGuo was observed after treatment with sevelamer, and the inflammatory factors IL-2 and IL-6 also tended to decrease (Brønden et al., 2020). Biguanides and sulfonylureas have been used to lower blood sugar, like metformin, glimepiride, pioglitazone and dapagliflozin. In clinical studies, hypoglycemic drugs can reduce the level of oxidation *in vivo* while lowering blood glucose (Sova et al., 2013; Wang et al., 2013; Shigiyama et al., 2017).

Environmental factors are also important influencing factors for oxidation of organisms. Previous studies have found that Mediterranean diet and carbohydrate-reduced high-protein dietary intervention can also reduce the level of oxidation in the body (Gutierrez-Mariscal et al., 2012; Skytte et al., 2020). Consumption of Green tea catechin, watermelon powder, and supplementation of paricalcitol and l-arginine also reduced oxidative damage levels (Oyama et al., 2010; Glenn et al., 2018; Fan et al., 2019). Although exercise can increase the body’s energy metabolism, produce more potential oxidation factors. Clinical and animal experiments have shown that physical exercise can increase the activity of antioxidant enzymes in cells and improve oxidative resistance (Radak et al., 2007; Tweedie et al., 2011; Villaño et al., 2015; Franzke et al., 2018; Larsen et al., 2020).

As we all know, T2D can cause many complications, and the mortality rate of cardiovascular disease is very high. Shokri suggested that increasing PON1 expression can reduce plasma oxidized-LDL level, reduce the ability of macrophages to absorb oxidized-LDL, and reduce cardiovascular complications in T2D patients. Therefore, the strategy of raising or restoring PON1 level is useful for reducing or preventing cardiovascular complications (Shokri et al., 2020).

## Conclusion

Oxidative stress has been advocated as an essential pathological factor for many diseases, especially in aging and neurodegenerative disorders. As noted above that pancreatic islets and pancreatic B cells have a high metabolic reaction and a low expression of antioxidant substances, so they are vulnerable to oxidative free radicals (Shah et al., 2007). As noted above that the oxidative stress in pancreatic B cells could reduce insulin synthesis, even make the cell prone to apoptosis (Keane et al., 2015; Poulsen et al., 2019). Furthermore, oxidative stress can lead to insulin resistance by disturbing the insulin receptor signaling pathway. Some researchers reported that oxidative stress can activate the proinflammatory signaling pathways of NF-kB and c-Jun N-terminal protein kinase, which can cause serine hyperphosphorylation in insulin receptor substrates (such as IRS1 and IRS2) (Evans et al., 2005; Shah et al., 2007; Xie et al., 2019). This may also suppress the function of GLUT-4 (Hurrle and Hsu, 2017).

Watson proposed T2D as a redox disease (Watson, 2014). In the redox research, DNA oxidation has been attracted more attention in the past few decades whereas a less focus on RNA oxidation. However, RNA oxidation has become the focus of current research because of its role in many diseases. RNA are more susceptible to oxidation than DNA (Hofer et al., 2006), which may be inferred from the following observations. First, its single-stranded nature and widely distributed around the mitochondria that produce reactive oxygen species, making it vulnerable to free radical (Hofer et al., 2006; Nunomura et al., 2012; Poulsen et al., 2012). Second, the repair mechanism of RNA activity is not fully understood, degradation and elimination are the main ways that are coping with RNA oxidative damage. Third, DNA is protected by proteins such as histones, and few proteins bind to RNA (Hofer et al., 2006; Nunomura et al., 2012; Poulsen et al., 2012).

8-oxoGuo is one of the products of RNA oxidation, so the actual oxidation levels in the body would be higher than measured. In the previous studies, 8-oxoGuo has the potential role of diagnostic detection, prognosis, and treatment target. Although there is strong evidence that oxidative stress has pathophysiological effects and the antioxidant effects of existing diabetes-related treatments have been proposed, however, the clinical outcomes of antioxidant trials have been disappointing. Many drugs cannot cure T2D fundamentally, they can only relieve symptoms. Recently studies have found some drugs can reduce the oxidation of T2D and reduce the incidence of complications, but its mechanism is unclear and further studies are still required to determine causality in T2D patients. Increasing intracellular antioxidant defense and controlling the production of free radicals may be helpful to the treatment of T2D, like developing the new compounds that inhibiting the reactive oxygen-producing enzymes. In this review, we summarize the consequences and cellular handling mechanisms of the oxidative RNA damage that may provide new insights to the pathogenesis of T2D and lead to a better therapeutic strategy.
